# Choroidal Vascularity Index as a Biomarker for Visual Response to Antivascular Endothelial Growth Factor Treatment in Diabetic Macular Edema

**DOI:** 10.1155/2021/3033219

**Published:** 2021-11-26

**Authors:** Ningxin Dou, Shanshan Yu, Ching-Kit Tsui, Boyu Yang, Jianqiang Lin, Xi Lu, Yue Xu, Benjuan Wu, Jinfeng Zhao, Xiaoling Liang

**Affiliations:** State Key Laboratory of Ophthalmology, Zhongshan Ophthalmic Center, Sun Yat-sen University, Guangzhou 510060, China

## Abstract

**Purpose:**

To investigate the choroidal vascularity index (CVI) as a prognostic factor for the visual efficacy of antivascular endothelial growth factor (VEGF) treatment in diabetic macular edema (DME).

**Methods:**

We retrospectively reviewed 92 DME eyes receiving anti-VEGF treatment, which were stratified as responders (≥5 letters gained) and nonresponders (<5 letters gained or lost). Baseline systematic features and optical coherence tomography features, including the CVI, adjusted ellipsoid zone (EZ) reflectivity, subretinal fluid (SRF), and disorganization of the retinal inner layers (DRIL), were evaluated between the two groups.

**Results:**

The baseline CVI was significantly lower in nonresponders than in responders (0.66 ± 0.05, 0.69 ± 0.05, and 0.72 ± 0.05, *p* = 0.014). After adjusting for other factors, the baseline CVI, DRIL, SRF, and adjusted EZ reflectivity were significantly associated with visual outcomes (CVI: odds ratio (OR) = 0.17, *p* = 0.006; adjusted EZ reflectivity: OR = 0.56, *p* = 0.007; DRIL: OR = 6.71, *p* = 0.001; and SRF: OR = 0.29, *p* = 0.008).

**Conclusion:**

DME patients with a higher CVI, higher adjusted EZ reflectivity, the presence of SRF, and the absence of DRIL at baseline were more likely to gain >5 letters in visual acuity after anti-VEGF treatment. CVI may serve as a novel biomarker for visual response to anti-VEGF treatment in DME.

## 1. Introduction

Diabetic macular edema (DME) is a leading cause of visual impairment in patients with diabetes [1]. Intravitreal vascular endothelial growth factor (VEGF) inhibitors have achieved favorable functional and structural outcomes in patients with DME. In the RIDE and RISE study, 33.6%–45.7% of patients with DME had a best-corrected visual acuity (BCVA) gain of more than three lines after 2 years of anti-VEGF treatment. A considerable proportion of patients (19.7%–39.2%) responded poorly to treatment [2]. Moreover, repeated injections are often required to maintain the treatment efficacy for DME [3]. To assist therapeutic selection for individual patients with DME, identification of prognostic factors for visual response before anti-VEGF treatment is critically important.

Spectral-domain optical coherence tomography (SD-OCT), which provides a noninvasive retinal image, is undoubtedly a potential tool for this purpose. Previous studies have reported a series of OCT retinal morphological features associated with the visual prognosis of anti-VEGF treatment in DME, such as disorganization of the retinal inner layers (DRIL), disruption of the ellipsoid zone (EZ) and external limiting membrane (ELM), subretinal fluid (SRF), epiretinal membrane (ERM), and hyperreflective foci (HRFs) [4–8]. However, most studies have focused on retinal features in OCT, neglecting the prognostic value of choroidal features.

The choroidal vasculature provides oxygen and nutrients to the outer retina and supports highly metabolically active photoreceptors, particularly in the foveal region. Damage to the choroidal vasculature may cause severe functional damage to the retina and impair visual recovery. Choroidal abnormalities are considerable in DME pathology [9, 10]. Recognizing choroidal features may provide a more comprehensive picture of the prognostic factors for anti-VEGF treatment in DME. Enhanced depth imaging OCT (EDI-OCT) shows a more detailed and deepened choroidal vasculature image. Based on the EDI image, the newly developed marker of the choroidal vascularity index (CVI) provides information on the relative change between the stromal and luminal vascular components and is more robust than the subfoveal choroidal thickness (SFCT) [11]. The CVI shows a good correlation with the severity of diabetic retinopathy (DR) and decreases significantly in DME, which may be a sensitive marker of choroidal vascular change in DME [12, 13]. However, the association between the CVI and visual outcomes of anti-VEGF treatment in DME remains unclear.

In this study, we aimed to investigate the correlation between the CVI and visual prognosis of anti-VEGF treatment in DME. We performed multiple factor analysis to integrate the CVI and other candidate choroidal and retinal features to identify biomarkers that may provide more accurate prognostic information for anti-VEGF treatment in DME.

## 2. Materials and Methods

All research and measurements adhered to the tenets of the Declaration of Helsinki after the study protocol were approved by the Ethics Committee of Zhongshan Ophthalmic Center, Sun Yat-sen University. All participants provided written informed consent before enrollment in the study.

We conducted a retrospective case-control study. The recruited patients received three or more monthly intravitreal anti-VEGF drug injections (ranibizumab or conbercept) for the treatment of DME at Zhongshan Ophthalmic Center, Sun Yat-sen University, from February 2019 to July 2020. The patients had at least 4 months of follow-up. Patients who fulfilled the following criteria were included in the study: (1) age ≥ 18 years; (2) type 1 or 2 diabetes mellitus; and (3) center-involved DME treated with anti-VEGF drugs, with a study eye baseline BCVA lower than 0.63 (20/32). For patients who received bilateral injections, both eyes were included. The exclusion criteria were as follows: (1) other concomitant ocular diseases that compromise VA or cause macular edema (refractive errors of more than ±6 diopters, glaucoma, uveitis, or retinal vein occlusion); (2) previous vitreoretinal surgery or cataract surgery within 3 months; (3) previous intravitreal anti-VEGF drug injections or dexamethasone implants at any time; and (4) low-quality OCT image in which firm identification of retinal and choroidal features is not feasible. Mild lens opacity is acceptable if there is no impact on OCT image quality.

Baseline ocular and systemic data were recorded at the first visit, including demographic data, duration of diabetes, history of hypertension, and type of retinopathy (nonproliferative or proliferative stage). For the first three loading doses administered at monthly intervals, a doctor reviewed the patients 1 month after each injection. After that, a repeat course of injections was given depending on the ophthalmologist's judgment at the follow-up visit, taking a 3 + PRN regimen described in the previous study as reference [14]. Examination of BCVA using ETDRS visual acuity charts, comprehensive ophthalmologic examination with a slit-lamp microscope, intraocular pressure (IOP) measurement, and OCT scanning was performed at the baseline and every follow-up visit. BCVA after treatment was recorded 1 month after the last injection during the follow-up period, which was the last follow-up. Patients were categorized according to their BCVA evolution from baseline and were stratified into two treatment response groups: responders (≥5 ETDRS letters gained) and nonresponders (<5 ETDRS letters gained or lost) [5].

### 2.1. Optical Coherence Tomography Analysis

OCT scans were obtained using SD-OCT (Heidelberg Spectralis, Heidelberg, Germany). Vertical and horizontal line scans dissecting the fovea were acquired using the 30-degree EDI mode. One hundred images were overlaid to create high-resolution images.

**Figure 1:**
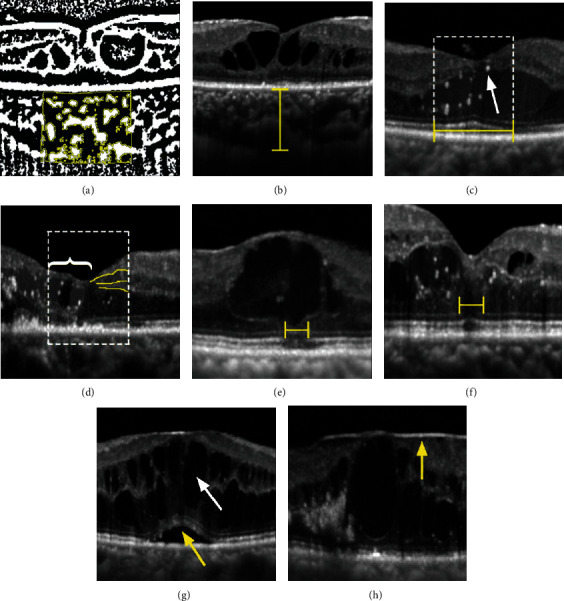
The morphological features evaluated in optical coherence tomography: (a) choroidal vascularity index (CVI); (b) subfoveal choroidal thickness (SFCT); (c) adjusted ellipsoid zone and external limiting membrane reflectivity; hyperreflective foci (HRF) (white arrow); (d) disorganization of the retinal inner layers (DRIL); (e) disruption of the ellipsoid zone; (f) disruption of the external limiting membrane; (g) the presence of subretinal fluid (SRF) (yellow arrow) and cystoid abnormalities (white arrow); (h) presence of an epiretinal membrane.

A series of qualitative and quantitative morphological features were evaluated in the baseline OCT images as shown in Figure 1, including features describing the choroid, as follows: (1) CVI and (2) SFCT. Additionally, the following features describing the retina were considered: (1) adjusted EZ reflectivity, (2) adjusted ELM reflectivity, (3) DRIL, (4) disruption of the EZ, (5) disruption of the ELM, (6) presence of SRF, (7) presence of cystoid abnormalities, (8) presence of an ERM, and (9) HRF quantity.

The listed features were evaluated on horizontal and vertical scans passing through the fovea. All image analyses were performed using ImageJ, a software program in the public domain.

The CVI was calculated using the protocol described by Sonoda et al. [15]. The subfoveal choroidal area of 1.5 mm wide, centered on the fovea, was selected as the total choroidal area (TCA). The luminal choroidal area (LCA) was obtained after Niblack's auto local binarization was applied to the OCT image. The CVI was calculated as the ratio of the LCA to TCA. The SFCT was measured as the distance between Bruch's membrane and the choroid-scleral interface just beneath the fovea.

Morphological features describing the retina were measured within the central 1 mm area in both vertical and horizontal scans. The OCT images were exported without adjustment to the preset contrast or brightness in the OCT software. The extent of EZ and ELM disruption was measured as the transverse length with loss of the hyperreflective signal that characterizes the EZ and ELM, respectively. The reflectivity is represented by the gray value. EZ, ELM, and RPE were outlined in the 1 mm zone, in which the average reflectivity was measured. Adjusted EZ and ELM reflectivity was calculated as raw data relative to the average reflectivity of the RPE. The results of the measurements on vertical and horizontal sectional images were averaged. DRIL was defined as the inability to distinguish boundaries between any two of the ganglion cell/inner plexiform layer complex, inner nuclear layer, and outer plexiform layer in >50% of the foveal 1 mm zone [4]. The presence of DRIL, SRF, cystoid abnormalities, and epiretinal membranes on either vertical or horizontal sectional images was recorded.

### 2.2. Statistics

To adjust for correlation between the two study eyes within the same patient, a generalized estimating equation (GEE) was used to compare the baseline ocular features between the responder and nonresponder groups. Odds ratios (ORs) with confidence intervals (95% CIs) were generated from the GEE-corrected binary logistic regression.

All tests were two-sided, and the significance level was set at 0.05. Statistical analyses were performed using IBM SPSS Statistics 20.

## 3. Results

A total of 97 eyes from 72 patients were included in the study. After excluding five DME eyes with low-quality OCT images, 92 DME eyes from 67 patients were included in the analysis. In the total 67 patients, the mean age was 57.9 ± 10.1 years. 41 patients (61.2%) were male. 38 patients (56.7%) had hypertension. 27 patients (40.3%) had histories of diabetes more than 10 years.

According to BCVA changes from baseline to 1 month after the last injection, 50 eyes (54.3%) were considered responders, whereas 42 eyes (45.7%) were considered nonresponders. The mean number of anti-VEGF injections during the follow-up period was 3.5 (range: 3–6). The mean duration of follow-up was 5.1 months (range: 4–8). Baseline ocular characteristics based on visual treatment responses are shown in Table 1. There were no significant differences in baseline ocular characteristics between the responders and nonresponders.

### 3.1. Baseline Optical Coherence Tomography Morphological Features between Responders and Nonresponders

**Figure 2:**
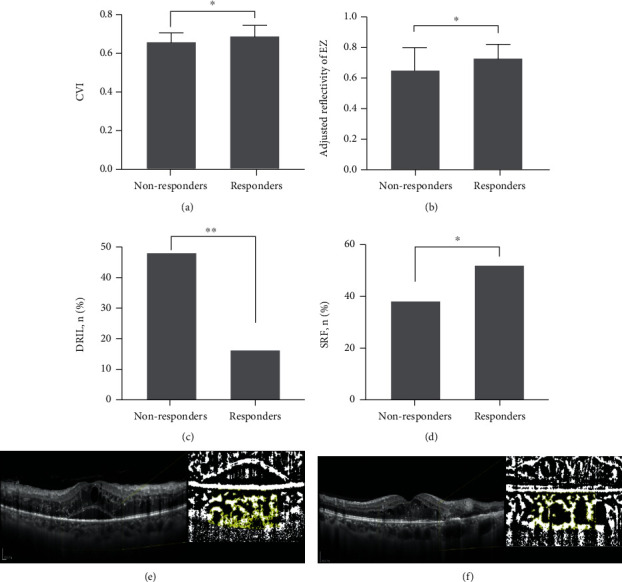
Baseline optical coherence tomography (OCT) morphological features between responders and nonresponders. Baseline choroidal vascularity index (CVI) (a). Adjusted ellipsoid zone (EZ) reflectivity (b). Proportion of disorganization of the retinal inner layers (DRIL) (c). Subretinal fluid (SRF) (d) in responders and nonresponders. The left eye of a 50-year-old man had a baseline CVI of 62%. Her baseline BCVA was 20/100. After three injections of VEGF inhibitors, the visual acuity decreased to 20/160. The treated eyes were categorized as nonresponders (e). The right eye of a 44-year-old man had a baseline CVI of 69%. Her baseline BCVA was 20/63. After three injections of VEGF inhibitors, the visual intensity increased to 20/40. The treated eyes were categorized as responders (f).

The comparisons of the baseline OCT structural characteristics are shown in Table 2. The baseline CVI was significantly different between the two groups. Responders exhibited significantly higher CVI than nonresponders at baseline (0.69 ± 0.06 vs. 0.66 ± 0.05, *p* = 0.014, Figure 2(a)). This suggests that the baseline CVI may be related to the visual prognosis of DME anti-VEGF treatment. Compared with the nonresponder group, the baseline LCA and TCA of the responders were larger; however, the differences were not significant. However, as measured by the percentage of the difference in the mean value of the responder group, the degree of difference in LCA between the two groups was greater than that of TCA (13% vs. 9%). This indicated that the higher baseline CVI in responders may be more affected by the difference in LCA. There was no statistically significant difference in the SFCT between the two groups.

Analysis of the retinal characteristics of the OCT showed that the baseline adjusted EZ reflectivity in the responder group was significantly higher than that in the nonresponder group (0.73 ± 0.09 vs. 0.65 ± 0.15, *p* = 0.006, Figure 2(b)). Notably, there was no statistical difference in the range of EZ interruption between the two groups, and the standard deviation of the results was large, reflecting large individual differences. In the baseline OCT, the proportion of DRIL in the nonresponders was significantly higher than that in responders (47.6% vs. 16.0%, *p* = 0.001, Figure 2(c)), suggesting that DRIL may be related to the poor response after DME treatment. The proportion of SRF in the responders was significantly greater than that in nonresponders (52.0% vs. 38.1%, *p* = 0.038, Figure 2(d)), suggesting that baseline SRF may be a protective factor for visual improvement after anti-VEGF treatment. No relationship was found between the treatment response and the adjusted ELM reflectivity, disruption of the EZ or ELM, cystoid abnormalities, ERM, or HRF quantity.

In conclusion, compared with the nonresponders, the responder group had a high mean baseline CVI and adjusted EZ reflectivity, a high proportion of SRF, and a low proportion of DRIL.

### 3.2. Multifactor Regression Analysis for Detecting the Prognostic Factors of the Response to the Anti-VEGF Treatment

Several OCT characteristics, including the CVI that showed significant differences between the two groups, were found through the above analysis. A GEE-corrected binary logistic regression was performed to further identify the independent prognostic factors for the visual response after anti-VEGF treatment, including age, baseline BCVA, stage of retinopathy, the CVI, adjusted EZ reflectivity, the presence of DRIL, and the presence of SRF. The results are shown in Table 3. The higher CVI at baseline seems to be a protective factor for BCVA gain from anti-VEGF treatment, with an OR of 0.17 (*p* = 0.006) for nonresponders versus responders. Alternatively, for every 0.1 increase in the baseline CVI, the risk of being nonresponders after anti-VEGF treatment was reduced by 83%. The higher adjusted EZ reflectivity and presence of SRF at baseline were also protective for the visual response to anti-VEGF treatment. Similarly, for every 0.1 increase in baseline EZ relative light reflectance, the risk of poor response after anti-VEGF treatment was reduced by 44% (OR = 0.56, *p* = 0.007). The risk of poor response after anti-VEGF treatment in DME patients with SRF at baseline OCT was 29% of patients without SRF (OR = 0.29, *p* = 0.008). The presence of DRIL at baseline was a risk factor for poor visual response to treatment, with ORs of 6.71 (*p* = 0.001).

Before treatment, DME patients with high CVI and adjusted EZ reflectivity, SRF, and no DRIL were likely to gain visual acuity improvement of 5 letters or more.

## 4. Discussion

In this study, we identified CVI as a novel biomarker, along with DRIL, SRF, and adjusted EZ reflectivity, which may predict the visual response to anti-VEGF treatment. A higher CVI, the absence of DRIL, the presence of SRF, and higher adjusted EZ reflectivity at baseline indicated that the individual was more likely to be a responder and gain more than five letters in BCVA after anti-VEGF treatment.

In recent years, SFCT has been commonly used to evaluate the choroid in DR or DME. However, SFCT is an unstable factor affected by various systemic and ocular factors, such as age, axial length, IOP, or systolic blood pressure, which was reflected in the inconsistent results of SFCT in DR or DME in different studies. SFCT has been reported to be reduced, unchanged, and even increased in DR [16, 17]. The relationship between baseline SFCT and the treatment response of VEGF in DME has been variable in previous studies. Rayess et al. found that a greater baseline SFCT was associated with a better anatomic and functional response [18], whereas Campos et al. showed that SFCT did not predict the DME outcome [19]. In this study, the SFCT exhibited a large degree of variation and was not significantly different between responders and nonresponders. Compared with SFCT, the CVI showed less variability under the above-mentioned physiological factors, indicating that it is a relatively stable choroidal index. Unlike SFCT, which works as a crude parameter, the CVI discriminates between the luminal and stromal areas, providing more detailed information about changes in the choroidal vessels. In our study, we found that the CVI in any group showed low variability, and the baseline CVI was significantly lower in nonresponders than in responders.

Changes in the CVI in DR have been reported in several recent studies. Gupta et al. reported that the CVI was significantly decreased in eyes with DME and DR compared to controls [12]. A decrease in the CVI was observed in patients with diabetes even in the absence of DR, and a further decrease occurred along with the severity of DR [13]. These results suggest that the CVI is a relatively early and sensitive biomarker of diabetic fundus changes that may predict DR development. In the current study, we reported the CVI as a possible novel biomarker for the functional efficacy of anti-VEGF treatment in DME for the first time. Considering that the severity of DR is associated with the CVI, we included the CVI, the severity of DR, and other possibly related baseline factors in the multivariate analysis; a high baseline CVI remained significantly related to a greater possibility of being a functional responder after treatment, which confirmed the predictive value of the CVI independent of factors including the severity of DR.

The choroid is important in the metabolic exchange of the outer layers of the retina, particularly the photoreceptors, which are critical for visual function. Histopathologic studies revealed variable choroidal vascular changes secondary to diabetes mellitus, such as vascular dropout, areas of vascular nonperfusion, and choroidal neovascularization [9]. The exact role of choroidal abnormalities in the pathology of DME and its impact on the response to anti-VEGF treatment remain unclear. Some early histopathologic and functional studies can provide insight into this subject. Studies from postmortem subjects demonstrated that diabetic choroids had a more than fourfold greater focal choriocapillaris degeneration area than nondiabetic choroids [20]. In addition to the choriocapillaris, large and medium choroidal vessels are also involved. Focal vascular loss in Sattler's layer, vascular stump, and narrowing in Haller's layer have been observed more frequently in patients with DR than those in controls [21]. Structural damage in the three layers of choroid vessels may affect choroidal blood flow. Laser Doppler flowmetry and OCT angiography measurements revealed that patients with diabetes, particularly those with DME, exhibited significantly decreased choriocapillaris blood flow in the foveal region [22, 23].

Choroidal vasculature impairment and associated decreased choriocapillaris blood flow can aggravate hypoxia of the outer retina, which increases the expression of VEGF and promotes the formation of macular edema [10]. Damage to the choriocapillaris and larger choroidal vessels involved in diabetes, as described above, may cause a decrease in the CVI. Choroidal blood flow, which partly reflects the choroidal vascular perfusion state, may also involve changes in the CVI. It is hypothesized that DME eyes with a low CVI at baseline may already have relatively severe structural damage of choroidal vessels, which cannot be reconstructed during anti-VEGF treatment. This group of patients may also present a relatively obvious decrease in the choroidal blood flow at baseline, which may hinder the absorption of edema liquid. These two aspects of choroidal vascular changes may result in the individual eye with a low CVI being likely to be a nonresponder to anti-VEGF treatment. According to the hypothesis above, the change in the luminal area possibly played a major role in the difference in the CVI between the two groups, which was consistent with our results that the difference in LCA was greater than that of TCA between the two groups. Further studies are necessary to reveal the detailed pathophysiological mechanisms behind the change in the CVI and its relation to the response to anti-VEGF treatment.

DRIL on OCT was considered to indicate structural damage in the transmission pathway of visual signals from photoreceptors to retinal ganglion cells, which was correlated with a visual response to anti-VEGF treatment in eyes with DME [4]. Our results support the findings of previous studies that DRIL is a valuable predictive biomarker, with a risk for poor visual outcomes that is almost seven times higher than that in patients without DRIL.

According to our results, SRF appears to be a protective factor. The effects of SRF on treatment outcomes following anti-VEGF treatment have not been fully clarified. Our result is consistent with some of the previous studies that baseline SRF is related to a greater visual or structural improvement of anti-VEGF treatment [8, 24]. A possible reason is that compared to diffuse retinal thickening or cystoid macular edema, the internal structure of the neuroretina is less disturbed in SRF, which may result in less mechanical damage to retinal nerve cells. Some studies provide opposite conclusions. Seo et al. reported that SRD was correlated with poorer visual outcomes, and disruption of photoreceptor integrity occurred more frequently in SRD. Considering that the analysis was performed in a relatively small number of patients (16 patients in the SRD group) and simultaneous presence of diffuse retinal thickening or cystoid macular edema was also classified as SRD in this study, the conclusions need further demonstration [25].

In contrast to previous studies, we did not find significant associations between the extent of EZ or ELM disruption and the functional response to anti-VEGF treatment. This is partly because, in many cases of DME eyes, the EZ and ELM gradually lose hyperreflectivity in an inhomogeneous manner. Disruption of the EZ and ELM did not occur as a definite area. It is challenging to measure the extent of disruption precisely. From this consideration, the reflectivity of the whole area of the EZ and ELM in the central 1 mm zone may be a more representative marker for quantitative evaluation. Guyon et al. reported that EZ reflectivity was lower in resolved DME than that in diabetic eyes without DME and nondiabetic control eyes [26]. A larger proportion of EZ with reflectivity recovering to the normal level after 1 week can predict better BCVA treatment response at 1 month [27].

In our study, considering that the opacity of refracting media, such as cataract or vitreous conditions and differences in the ingredients of edema, affected the transmission of light, which altered the reflectivity of retinal layers on the light path, the adjusted reflectivity of EZ and ELM was used by calculating the ratio of the reflectivity of RPE on the same optical axis. In our study, the adjusted reflectivity of EZ at baseline was significantly lower in nonresponders than in responders. The EZ on OCT was supposed to represent a cellular compartment full of mitochondria in photoreceptors in histology. EZ reflectivity is closely correlated with the cone density [28]. Decreased reflectivity of EZ may be related to the loss of mitochondria, leading to dysfunction of photoreceptors or the direct loss of photoreceptors at baseline, resulting in a poor visual response. After adjusting in the multivariate analysis, the higher adjusted reflectivity of EZ was associated with a lower risk of being a visual nonresponder, which indicated that the adjusted reflectivity of EZ may also be a prognostic factor for anti-VEGF treatment in DME.

The limitations of this study include its retrospective design and relatively small sample size. BCVA after treatment was evaluated 1 month after the last injection. The number of injections differed among patients, ranging from three to six. Prospective studies with longer follow-up periods are needed to confirm these findings. Besides OCT exhibiting the structural features of the retina and choroid, functional examinations, such as OCTA, and intraocular inflammatory factors are also related to the efficiency of treatment [29]. An integrated prediction model should be developed to optimize patient care in the context of DME. In conclusion, this study demonstrated the CVI as a possible novel biomarker of visual response to anti-VEGF treatment. DRIL, SRF, and the adjusted reflectivity of EZ were also confirmed to be useful markers strongly associated with anti-VEGF treatment efficiency.

## 5. Conclusion

DME patients with a higher CVI, the absence of DRIL, the presence of SRF, and higher adjusted EZ reflectivity at baseline were more likely to gain more than five letters in BCVA after anti-VEGF treatment. The CVI is possibly a novel prognostic factor for anti-VEGF treatment.

## Figures and Tables

**Table 1:** Baseline ocular characteristic comparison between responders and nonresponders by a generalized estimating equation model with adjusting for correlation between two eyes within the same patient (*N* = 92 eyes).

	Responders (*n* = 50)	Nonresponders (*n* = 42)	*p*
OCT quality index	36.6 ± 4.6	36.1 ± 3.8	0.509
Number of injections	3.4 ± 0.8	3.7 ± 0.9	0.094
PDR (%)	28 (56.0)	26 (61.9)	0.300
BCVA	36.4 ± 16.2	41.4 ± 15.1	0.137

OCT: optical coherence tomography; PDR: proliferative diabetic retinopathy; BCVA: best-corrected visual acuity.

**Table 2:** Baseline optical coherence tomography morphological feature comparison between responders and nonresponders by a generalized estimating equation model with adjusting for correlation between two eyes within the same patient (*N* = 92 eyes).

	Responders (*n* = 50)	Nonresponders (*n* = 42)	*p*
LCA (mm^2^)	0.39 ± 0.12	0.34 ± 0.11	0.059
TCA (mm^2^)	0.56 ± 0.15	0.51 ± 0.15	0.168
SFCT (*μ*m)	365.85 ± 92.77	334.61 ± 92.00	0.145
CVI	0.69 ± 0.06	0.66 ± 0.05	0.014
Reflectivity of EZ	140.9 ± 25.3	129.0 ± 34.2	0.030
Reflectivity of ELM	94.2 ± 21.7	91.4 ± 28.7	0.538
Reflectivity of RPE	193.0 ± 18.2	197.5 ± 14.8	0.243
Adjusted reflectivity of EZ	0.73 ± 0.09	0.65 ± 0.15	0.006
Adjusted reflectivity of ELM	0.49 ± 0.10	0.46 ± 0.13	0.335
Disruption of the EZ (*μ*m)	91.22 ± 164.44	160.40 ± 234.30	0.168
Disruption of the ELM (*μ*m)	71.50 ± 170.60	105.88 ± 181.78	0.421
DRIL, *n* (%)	8 (16.0)	20 (47.6)	0.001
SRF, *n* (%)	26 (52.0)	16 (38.1)	0.038
Cystoid abnormalities, *n* (%)	39 (78.0)	33 (78.6)	0.987
Epiretinal membrane, *n* (%)	16 (32.0)	13 (31.0)	0.640
HRF	10.98 ± 9.03	10.70 ± 8.82	0.563

LCA: luminal choroidal area; TCA: total choroidal area; SFCT: subfoveal choroidal thickness; CVI: choroidal vascularity index; EZ: ellipsoid zone; ELM: external limiting membrane; RPE: retinal pigment epithelium; DRIL: disorganization of the retinal inner layers; SRF: subretinal fluid; HRF: hyperreflective foci.

**Table 3:** Multifactor regression analysis for detecting the prognostic factors of the response to the antivascular endothelial growth factor (VEGF) treatment by a generalized estimating equation model with adjusting for correlation between two eyes within the same patient (*N* = 92 eyes).

	OR (95% CI)	*p*
Age	1.01 (0.97, 1.07)	0.658
BCVA	1.00 (0.97, 1.04)	0.840
Stage of DR	1.04 (0.41, 2.63)	0.942
CVI	0.17 (0.05, 0.61)	0.006
Adjusted reflectivity of EZ	0.56 (0.36, 0.86)	0.007
DRIL	6.71 (2.26, 19.89)	0.001
SRF	0.29 (0.12, 0.73)	0.008

OR: odds ratio; CI: confidence interval; BCVA: best-corrected visual acuity; CVI: choroidal vascularity index; EZ: ellipsoid zone; DRIL: disorganization of the retinal inner layers; SRF: subretinal fluid.

## Data Availability

The data used to support the findings of this study are available from the corresponding author upon request.
